# How the wild things are: comparative T cell phenotyping and memory T cell identification in wild boar and domestic pigs

**DOI:** 10.3389/fimmu.2026.1844742

**Published:** 2026-06-18

**Authors:** Alexander Schäfer, Virginia Friedrichs, Paul Deutschmann, Claudia Klein, Sandra Blome, Ulrike Blohm

**Affiliations:** 1 Institute of Diagnostic Virology, Friedrich-Loeffler-Institut, Federal Research Institute for Animal Health, Greifswald-Insel Riems, Germany; 2 Friedrich-Loeffler-Institut, Institute of Farm Animal Genetics, Neustadt, Germany; 3 Institute of Immunology, Friedrich-Loeffler-Institut, Federal Research Institute for Animal Health, Greifswald-Insel Riems, Germany

**Keywords:** adaptive memory, comparative immunology, domestic pig, immunity, T cells, wild boar

## Abstract

Domestic pigs constitute a significant global livestock resource and are increasingly recognized as valuable biomedical models, enabling fundamental research. In contrast, immunological studies on wild boar remain limited. To address this knowledge gap, we conducted a comparative analysis of baseline immune phenotypes in age-matched cohorts of domestic pigs and wild boar. Additionally, we adapted a staining and flow cytometry gating strategy based on human immunophenotyping protocols to define conventional memory T cell subsets in both species, validating this approach through proliferation assays and cytokine expression profiling. Our results demonstrated that wild boar exhibit a decreased frequency of polymorphonuclear neutrophils and natural killer cells in peripheral blood. Swine Leukocyte Antigen class II (SLA-DR) expression was significantly reduced across all professional antigen-presenting cell populations. Furthermore, the proportion of T lymphocytes was elevated in wild boar relative to domestic pigs. The T cell compartment in wild boar was characterized by a pronounced bias toward γδ T cells, accompanied by reduced frequencies of differentiated CD4^+^CD8α^+^ αβ T cells and late-stage memory T cells. Notably, these cells still expressed markers indicative of high activation. Our findings highlight intrinsic immunological differences between domestic pigs and wild boar and provide a foundational framework for future functional immunological studies, particularly in the context of infectious disease research and immunization strategies.

## 1 Introduction

Domestic pigs (*Sus scrofa domesticus*) represent a major and steadily increasing part of the global livestock population. Given key similarities in their genomic and immunologic architecture to the human immune system, they are also increasingly used as an additional model species for infections and immunizations (1–6). Their wild conspecific in the *Sus* genus, the Eurasian wild boar (*Sus scrofa scrofa*), is economically less important but host for a plethora of bacterial and viral pathogens as well (7). The spread of important veterinary pathogens like African swine fever virus (ASFV) is coupled to wild boar populations, and pathogen introduction into rare and endangered pig species or domestic pig holdings represent a major threat for animal conservation efforts and economic interests alike (8–10). Therefore, efficacious vaccines are necessary for animal welfare and disease control. However, these approaches require extensive knowledge of the porcine immune system, which is still lacking in many aspects for domestic pigs and is even more limited for wild boar.

The few previous studies comparing domestic pigs and wild boar showed increased mortality in wild boar even after infection with moderately virulent ASFV (16, 17), as well as divergent T cell responses after infection with both moderately and highly virulent ASFV (18, 19). These studies also found more pronounced proinflammatory responses in wild boar, like induction of CD8α^+^ cytotoxic and T-bet-dependent T cell responses, as well as more distinct γδ T cell responses (18, 19). The latter correlated with tissue damage and Kupffer cell degeneration in the liver (20). However, systematic analyses of the immune system baselines of healthy domestic pigs and wild boar are missing. Comparative studies in another set of conspecifics, laboratory and wild rodents, found considerable differences in immune composition and effector functions, with wild animals commonly showing increased activation but attenuated antigenic responses, at least to *in vitro* stimulation (11–15). Whether similar differences are present between domestic pigs and wild boar, remains unknown. However, if composition and effector functions of the immune systems of domestic pigs and wild boar are inherently different, they likely also require targeted or species-specific approaches for various interventions, especially vaccination campaigns.

A central aspect of immunological studies in the context of vaccination against and infection with ASFV is the investigation of T cell memory responses (21). However, differences in the phenotype of memory T cells impairs comparative approaches between species. Memory T cells in mice are identified by differential expression of CD44 and CD62L (L-selectin) (22), which is not transferable to pigs because of high CD44 expression levels regardless of activation on porcine lymphocytes (23). The approach for murine cells is also substantially different to human memory T cells, which are usually differentiated based on expression of CD45RA, CD27, and CCR7 or CD62L. This combination allows for identification of naïve T cells (CD45RA^+^CD27^+^CCR7^+^), early effector T cells (Eff, CD45RA^+^CD27^+^CCR7^−^), central memory T cells (CM, CD45RA^−^CD27^+^CCR7^+^), effector memory T cells (EM, CD45RA^−^CD27^+/−^CCR7^−^), and terminally differentiated effector memory T cells with re-expression of CD45RA (EMRA, CD45RA^+^CD27^−^CCR7^−^) (24–27). Additional studies of human lymphocytes have shown synchronized expression of CD62L and CCR7 on memory subsets (28). Several additional subpopulations in humans, like stem cell-like (29) or virtual (30) memory T cells, have been proposed since, highlighting the complexity of adaptive cellular immunity.

Several studies investigated T cell memory responses in pigs but they usually focused on distinct cell subsets or employed a narrow set of markers. While CD8α^+^ αβ T cells have also been investigated recently (31), analysis of memory T cells in pigs often focused on responses of CD4^+^CD8α^+^ αβ T cells (32–37). However, focus on CD4^+^CD8α^+^ T cells harbors the risk of missing memory formation in other subsets. CD27 has been used as a marker for differentiation of porcine T cells, as loss of CD27 expression is coupled with progressing maturation in pigs (31, 38). Other studies have used combinations of CD8α and CCR7 (2), CD27 and CD11a (31), or CD45RA and CCR7 (39). These analyses are not harmonized and often done with reduced antibody panels, resulting in potential loss of comparability, resolution, and true subset identification. Therefore, while individual markers have been used in studies previously, a combined approach with systematic evaluation of these cell populations is missing.

To investigate the differences between domestic pigs and wild boar further and thus inform on the potential need for more targeted approaches in these species, we investigated samples from age-matched domestic pigs and wild boar in comparative assays employing multiparametric flow cytometry to systematically assess potential differences, with a focus on the T cell landscape. We propose an analysis platform for porcine memory T cells by multicolor flow cytometry, further validated by functional assays and marker expression analysis by PCR, that enables in-depth analysis of porcine T cell memory responses and direct comparisons with the human T cell memory landscape.

## 2 Materials and methods

### 2.1 Animals and biological samples

Wild boar were direct offspring from wild caught boar and kept under conventional conditions with outdoor contact at Friedrich-Loeffler-Institut (FLI), Germany. They were fed conventional pig feed mixed with fruit, vegetables, meat, and fish. The 13 animals (6 female, 7 male) in this study were approximately four months of age. Ten age-matched domestic pigs (5 female, 5 male) were conventionally kept at FLI and fed exclusively with conventional pig feed. Domestic pigs (German Landrace) were vaccinated against porcine circovirus 2 and mycoplasma after weaning, wild boar were not vaccinated. Prior to sampling, juvenile wild boar were sedated via darting (3 ml dart syringe with a 1.2 × 38 mm needle, Telinject) with a blow pipe (B31.C, Telinject, length 1 m, caliber 11 mm) using Zoletil 100 (tiletamine, zolazepam; Virbac) at 3.3 mg/kg. Blood for *in vitro* studies was taken from naïve animals kept in a conventional holding facility at FLI. All samples were taken during summer (July). All European and national animal welfare guidelines were followed and sampling was conducted according to current German animal welfare regulations and approved by the local competent authority, LALLF-MV (Landesamt für Landwirtschaft und Fischerei, Mecklenburg-Vorpommern), under file reference 7221.3-1-01/23.

### 2.2 Cell isolation and culture

Cells were isolated and cultured as described previously (5). Briefly, peripheral blood mononuclear cells (PBMC) were isolated by density gradient centrifugation using Pancoll (PAN-Biotech, Germany) for 30 min at 760 × g and room temperature. Afterwards, cells were washed once with PBS-EDTA (1 mM) for 10 min at 350 × g and 4 °C, counted using a Neubauer improved haemocytometer, and used for flow cytometry or *in vitro* assays. Unless stated otherwise, cells were cultured in Ham’s F12/IMDM (1:1), supplemented with 10% FCS, 2-mercaptoethanol (50 μM), 100 U/ml penicillin, and 100 μg/ml streptomycin. If cells were not used immediately, they were frozen in cell culture media supplemented with 30% fetal calf serum (FCS) and 10% DMSO.

Freshly isolated or thawed PBMCs were either used directly for flow cytometric-based assays or cultured for analysis of proliferative responses and cytokine expression. For proliferation assays, PBMCs were incubated with increasing concentrations of Concanavalin A (ConA, 0.01-10 µg/ml) or *Staphylococcal enterotoxin B* (SEB, 1–1000 ng/ml) for five days at 38.5 °C and 5% CO_2_. For cytokine responses, PBMCs were either rested overnight and then stimulated with ConA (10 µg/ml), SEB (100 ng/ml) for four hours or stimulated overnight with restimulation for the last four hours in the morning. Phorbol-12-myristate-13-acetate/ionomycin (PMA/Iono, 50 ng/ml and 1 µg/ml, respectively) was added as a separate positive control for the last four hours of incubation. To inhibit cytokine secretion, Brefeldin A (10 µg/ml) was added during the last four hours of incubation.

### 2.3 Flow cytometry and sorting

Freshly isolated or thawed PBMCs were stained with antibodies listed in **Table 1**. Incubation steps with antibodies for surface markers were carried out for 15 min at 4 °C in the dark. After completing surface staining, cells were fixed using Fixation buffer (Biolegend, USA) according to the manufacturer’s instructions. For intracellular targets, cells were further permeabilized using Intracellular Staining Permeabilization Wash Buffer (Biolegend, USA). Intracellular markers were stained for 30 min at room temperature in the dark. Cells were acquired either directly or on the next morning at the latest. At least 10^5^ single, live cells were analyzed for every sample. Cells were analyzed using either BD LSRFortessa (equipped with four lasers (UV 355 nm, Violet 405 nm, Blue 488 nm, Red 633 nm) and 15 fluorescence detection channels (UV 2, Violet 6, Blue 4, Red 3)) or BD Symphony A3 (equipped with five lasers (UV 355 nm, Violet 405 nm, Blue 488 nm, Yellow-Green 560 nm, Red 633 nm) and 28 fluorescence detection channels (UV 7, Violet 8, Blue 6, Yellow-Green 4, Red 3)) with BD FACS DIVA Software (all BD Biosciences, USA).

**Table 1:** Antibodies used in this study.

Method	Antigen	Clone	Conjugate	Isotype	Source	Catalog#	RRID
Flow Cytometry	CD3ϵ	PPT3	APC	M IgG1	Southern Biotech	4510-11	AB_2796016
	CD45RA	MIL13	FITC	M IgG1	Bio-Rad	MCA1751F	AB_323348
	CD8α	76-2-11	PE	M IgG2a	Southern Biotech	4520-09	AB_2796033
	CD4	74-12-4	PerCP	M IgG2b	BD Biosciences	561474	AB_10683310
	CCR7 (CD197)	3D12	BV421	R IgG2a	BD Biosciences	740052	AB_2739819
	L-Selectin (CD62L)	SK11	BUV805	M IgG2a	BD Biosciences	749209	AB_2873587
	CD27	b30c7	—	M IgG1	in-house	—	—
	γδ-CD3	PPT16	—	M IgG2b	in-house	—	—
	α-IgG1	A85-1	BUV395	R IgG	BD Biosciences	740234	AB_2739982
	α-IgG2b	polyclonal	PE-Cy7	G IgG	Southern Biotech	1090-17	AB_2794529
	α-IgG2b	R12-3	BV786	R IgG	BD Biosciences	743179	AB_2741330
	Ki-67	B56	BV786	M IgG1	BD Biosciences	563756	AB_2732007
	IFNγ	P2G10	PE	M IgG1	BD Biosciences	559812	AB_397341
	TNFα	Mab11	PE-Cy7	M IgG1	BD Biosciences	557647	AB_396764
Sorting (PCR analysis)	CD3ϵ	PPT3	APC	M IgG1	Southern Biotech	4510-11	AB_2796016
	CD45RA	MIL13	FITC	M IgG1	Bio-Rad	MCA1751F	AB_323348
	L-Selectin (CD62L)	SK11	BUV805	M IgG2a	BD Biosciences	749209	AB_2873587
	CD27	b30c7	—	M IgG1	in-house	—	—
	γδ-CD3	PPT16	—	M IgG2b	in-house	—	—
	α-IgG1	A85-1	BUV395	R IgG	BD Biosciences	740234	AB_2739982
	α-IgG2b	poly	PE-Cy7	G IgG	Southern Biotech	1090-17	AB_2794529

M, mouse; R, rat; G, goat.

Gating is shown in **Supplementary Figure 1**. Briefly, lymphocytes were identified according to their FSC/SSC characteristics. Doublets were excluded by consecutive FSC-W vs. FSC-H and SSC-W vs. SSC-H gating. Dead cells were identified by Zombie Aqua Fixable Viability Dye (Biolegend, USA) and excluded from analyses.

For sorting of cells for PCR analyses, freshly isolated PBMC of three naïve domestic pigs were first stained with a condensed antibody panel stated in **Table 1**. Stained PBMC were filtered using tubes with 35 µm cell strainer caps (Corning, USA) prior to sorting. Cells were sorted using a BD FACSAria Fusion (BD Biosciences, USA) with the 100 µm nozzle, 4-way purity setting, and low flow rate into FCS-pretreated Protein LoBind tubes (Eppendorf, Germany). At least 10^5^ cells were sorted for each condition. Sort quality was checked by flow cytometry (see **Supplementary Figure 3**) and confirmed at least 90% purity for all samples. Sorted cells were centrifuged and immediately lysed in Trizol.

Flow cytometric data was analyzed by FlowJo v10.10 (BD Life Sciences, USA) with the AutoSpill plugin used for compensation of multiparametric data (40).

### 2.4 Marker expression analysis by PCR and qRT-PCR

To verify cell identity with markers that are not detectable by flow cytometry in pigs, we applied PCR and quantitative real-time PCRs (qRT-PCR). Primers were designed using PrimerQuest (Integrated DNA Technologies, USA), ensuring primer lengths of 17–30 bp and GC content of 40-55%. Hairpin structures and high possibility of homo- or heterodimer formation were excluded by OligoAnalyzer Tool (Integrated DNA Technologies, USA). Finally, all sequences were blasted using NCBI BLAST tool to verify specific transcripts, which were experimentally verified by detection of PCR products at the expected amplicon size in absence of unspecific bands (for PCR products) and by melting curve analysis (for qRT-PCR products). Primer-specific annealing temperatures were determined by gradient PCR. Primer sequences and specifications are listed in **Table 2**.

**Table 2:** Primer sequences and specifications.

Method	Gene symbol	Gene name	Gene ID	Primer sequences [5’ 3’]	Amplicon size [bp]	Ref
Gel‐based PCR	*BACH2*	BTB domain and CNC homolog 2	100620522	F - CAAGCACATTCGGTGAAGATAAC R - AACAGGGTGAGGAGGAGAA	900	(43, 44)
	*CCL5*	C-C motif chemokine ligand 5	396613	F - GCCCTTGCTGTCATCCTC R - CCGCACCCATTTCTTCTCT	228	(45)
	*CCR6*	C-C motif chemokine receptor 6	100624245	F - TGCTCTTCGGCTTCTTCATC R - CACACACCACAGGTCTTTCA	356	(28, 46, 47)
	*CCR7*	C-C motif chemokine receptor 7	396663	F - ATGAGGTCACGGACGACTA R - GATGATGACGAGGTAGCAGAAG	665	(45)
	*CD27*	CD27 molecule	100520023	F - GAAAGGCTGCTCGCTGT R - GCTGGACCATCACACTCTG	202	
	*EEF1A1*	Eukaryotic translation elongation factor 1 alpha 1	574059	F - TTTGCTGAACTGAAGGAGAAGA R - CCAGAGGAGGATAGTCAGAGAA	158	(41, 42, 48)
	*GNLY*	Granulysin	396869	F - GCGACGGAGAGCAGTTC R - GGAGATGCGACGGAGAAAG	248	(45)
	*GZMB*	Granzyme B	100233184	F - CAACATCAAGAAACAGGAAGAGAC R - ATTAGGCAAGTAGGAGTCACAC	304	(45, 47)
	*HNRPLL*	Heterogeneous nuclear ribonucleoprotein L like	100622737	F - AGACCGAGGAGGGAGAAAT R - GGACAACAGGTGAAACAGAAAC	156	(47)
	*IFIT3*	Interferon induced protein with tetratricopeptide repeats 3	100154248	F - ACCAAACAATGGCTACCTCTATC R - CCTTTCCTTTCCTTCCTGTCTC	678	(45)
	*LEF1*	Lymphoid enhancer binding factor 1	100170126	F - ATCACACCCATCACACATCC R - TCTTGCTCCTTTCTCTGTTCAT	375	(49)
	*PRDM1*	PR/SET domain 1	100154284	F - GTGTGGTATTGTCGGGACTT R - GCTCTGGATTTGTGTGTCATTC	87	(47, 50–52)
	*SATB1*	SATB homeobox 1	100155111	F - TTACCAGGACGAAAGGGAAAG R - TGCCGTGATGCTTGAGATAG	776	(53)
	*SELL*	Selectin L	100127147	F - ATGGAACTGATTGCTGGACTTA R - GTCCTCCTTAGTCTTCCTGTTG	281	(45)
	*Tbx21*	T-box transcription factor 21	100518804	F - TCCCATTCCTGTCATTTACTGTG R - CCACTTGCCGCTCTGATAC	113	(45)
	*TCF7*	Transcription factor 7	100512951	F - GTCTACTCCGCCTTCAATCTG R - GCTTCTTGGCTTCCTTCTCT	538	(45, 49)
	*ZEB1*	Zinc finger E-box binding homeobox 1	100520325	F - CCCTCTCAACCTTTCCTCATC R - CACACAAATCGCAGGCATAC	611	(54)
	*ZEB2*	Zinc finger E-box binding homeobox 2	100739669	F - ACGAGAGGAAGAGGAAGATGA R - GTGTGAACTGTAGGAACCAGAA	745	(55, 56)
	*ZNF683*	Zinc finger protein 683	100524870	F - CTGAAGAAAGAGAACGGCAAGA R - GCAAGGCAAGCGAGAGAT	410	(57)
qRT‐PCR	*CD127* (*IL7R*)	C-C motif chemokine receptor 7	100271930	F - GCTGACGCTCCTACAAAGAA R - ACTCCAATCACTCCAGAAACC	106	(58)
	*CX3CR1*	C-X3-C motif chemokine receptor 1	100622126	F - CCTATCCATTCTCTACTGCCTTATTT R - CAGTGATGTTCTTGGGCTTCT	101	(59)
	*EEF1A1*	Eukaryotic translation elongation factor 1 alpha 1	574059	F - TTTGCTGAACTGAAGGAGAAGA R - CCAGAGGAGGATAGTCAGAGAA	158	(41, 42, 48)

Sorted cells were lysed in Trizol and RNA was isolated by phenol/chloroform method. cDNA was synthesized with LunaScript RT SuperMix Kit (New England BioLabs, UK) according to the manufacturer’s instructions. PCR reactions were done with the GoTaq G2 Flexi DNA Polymerase Kit (Promega, USA). For every reaction, 30 ng of template cDNA, 2 mM MgCl_2_, 5 mM dNTP (Jena Bioscience, Germany), 0.1 µM of each primer, and 1.25 U polymerase were mixed with 5X GoTaq G2 Flexi buffer. Nuclease-free water was added for a total volume of 25 µl. For qRT-PCR, the fluorescent dye EvaGreen (Jena Bioscience, Germany) was added. PCRs were run as follows: initial denaturation at 95 °C for 2 min, followed by 35 cycles (denaturation at 95 °C for 30 s, annealing at primer-specific temperature for 30 s, elongation at 72 °C for 30 s) and a final elongation step at 72 °C for 5 min. PCR products were visualized using 2.5% agarose gels with ethidium bromide staining (Carl Roth, Germany). PCR bands were analyzed using Image Lab software (Bio-Rad, USA). Band intensities were normalized to *EEF1A1*, which was selected as an internal reference gene based on previous validation as a suitable reference gene in porcine gene-expression studies in various cell types, including leukocytes (41, 42). Data were normalized for each marker for visualization.

### 2.5 Statistical analysis

Statistical analysis was performed using GraphPad Prism 10 (GraphPad Software Inc., Dotmatics, USA). Normality was confirmed by D’Agostino-Pearson test. Differences between two groups were tested by unpaired t-tests. Differences between more than two groups were tested by ordinary one-way ANOVA with Holm-Šídák’s *post-hoc* test. A *p* < 0.05 was considered statistically significant. Significant differences are indicated by their respective *p*-value, non-significant differences are not further designated. Effect sizes for all figures are shown in **Supplementary Tables 1**–**8**. Compact letter display was used to indicate differences between larger numbers of groups. Here, different letters indicate significant differences (*p* < 0.05), while identical letters indicate no significant differences. Unless specified otherwise, data are shown as mean (SD).

## 3 Results

### 3.1 The leukocyte landscape differs between domestic pigs and wild boar

To examine potential differences in the immune cell landscape of domestic pigs and wild boar, we utilized two cohorts of animals: Wild boar were bred from wild-caught animals and maintained in a stable with access to an outdoor environment. Age-matched domestic pigs (German Landrace) were kept under conventional indoor conditions.

**Figure 1:**
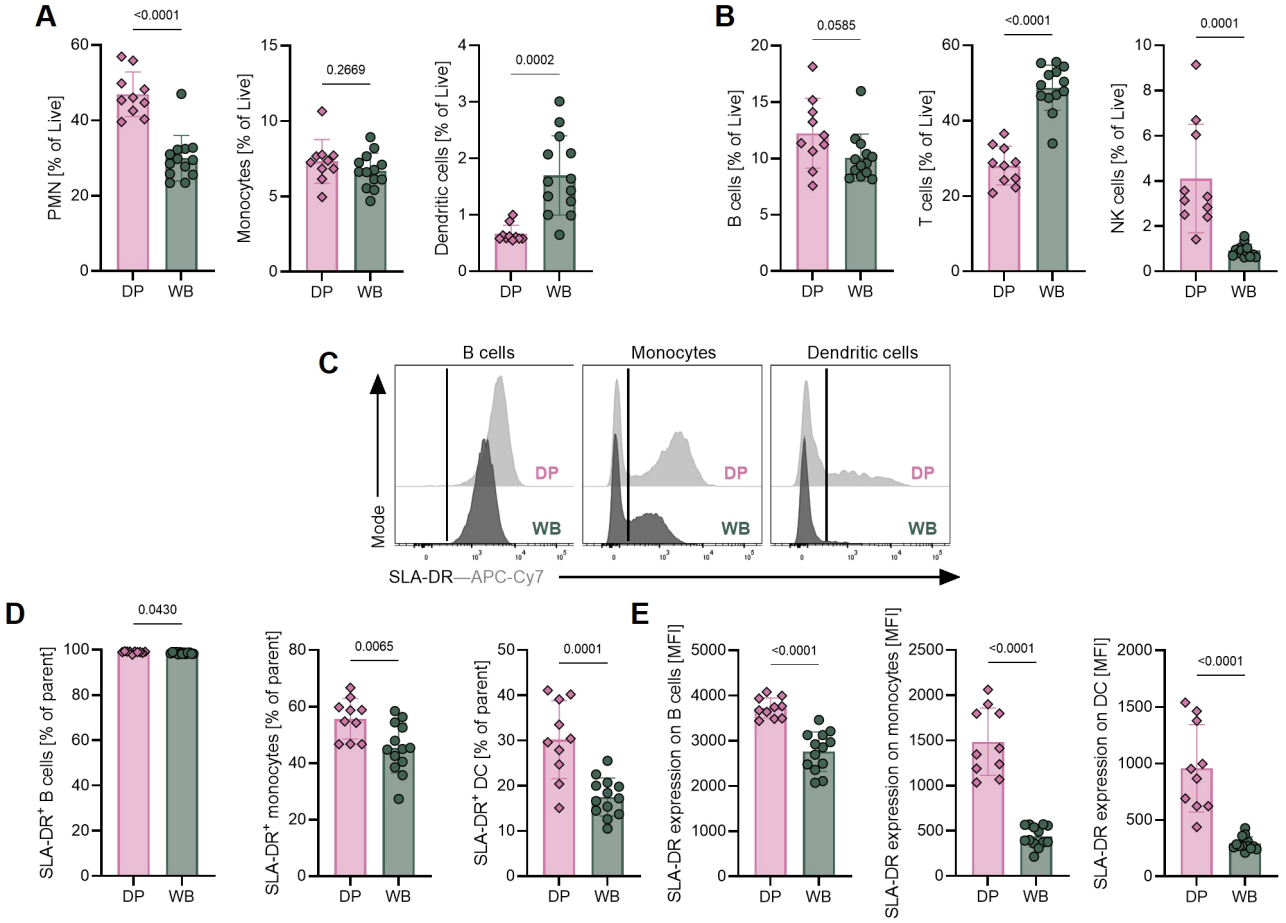
Comparative analysis of leukocyte populations in wild boar and domestic pig. Whole blood from domestic pigs (DP, *n* = 10, pink squares) and wild boar (WB, *n* = 13, green circles) was investigated by multiparametric flow cytometry. Bar graphs show frequencies of **(A)** myeloid (polymorphonuclear cells (PMN), monocytes, and dendritic cells (DC)) and **(B)** lymphoid (B cells, T cells, NK cells) populations in DP and WB. **(C)** Representative histograms show expression of SLA-DR on B cells (left), monocytes (middle), and DCs (right). Bar graphs show **(D)** frequencies of SLA-DR^+^ cells and **(E)** mean fluorescence intensity (MFI). Graphs show mean (SD). Statistical analysis by unpaired t-tests. *p* is indicated for all comparisons.

For a general overview of all leukocyte populations, whole blood was stained for multiparametric flow cytometry. After exclusion of debris, doublets, and dead cells (see gating strategy in **Supplementary Figures 1A, B**), myeloid cells were identified by expression of CD172a (Signal regulatory protein α, SIRPα) and further subdivided into polymorphonuclear cells (PMN), CD14^−^ dendritic cells (DC), and CD14^+^ monocytes. We found decreased frequencies of PMNs but increased frequencies of DCs in wild boar, while monocyte frequencies were similar between both species (**Figure 1A**). Lymphocytic cells were divided into CD79a^+^ B cells, CD3^+^ T cells, and CD3^−^CD8α^+^ NK cells. While B and NK cells were more frequent in domestic pigs, the T cell population was significantly larger in wild boar (**Figure 1B**).

We also investigated professional antigen-presenting cells, i.e., B cells, monocytes, and DCs, for expression of the class II major histocompatibility complex (MHC, swine leukocyte antigen, SLA, in pigs, SLA-DR). We found more SLA-DR^+^ cells and higher expression of SLA-DR in domestic pigs, especially on monocytes and DCs (**Figures 1C, D**).

### 3.2 Differentiated αβ and γδ T cells are less frequent but typically highly activated in wild boar

Further analyses were focused on the T cell compartment (see gating strategy in **Supplementary Figure 1C**). Instead of whole blood, we used freshly isolated PBMCs to investigate subpopulations and differentiation status. T cells were divided into conventional αβ T cells (CD3^+^γδCD3^−^) and γδ T cells (CD3^+^γδCD3^+^). αβ T cells were further divided according to their expression of CD4 and CD8α: CD4^+^CD8α^−^ T helper cells, CD4^−^CD8α^+^ cytotoxic T cells, and CD4^+^CD8α^+^ effector T cells. γδ T cells were investigated for CD8α expression: CD8α^−^ naïve γδ T cells and CD8α^+^ effector γδ T cells.

**Figure 2:**
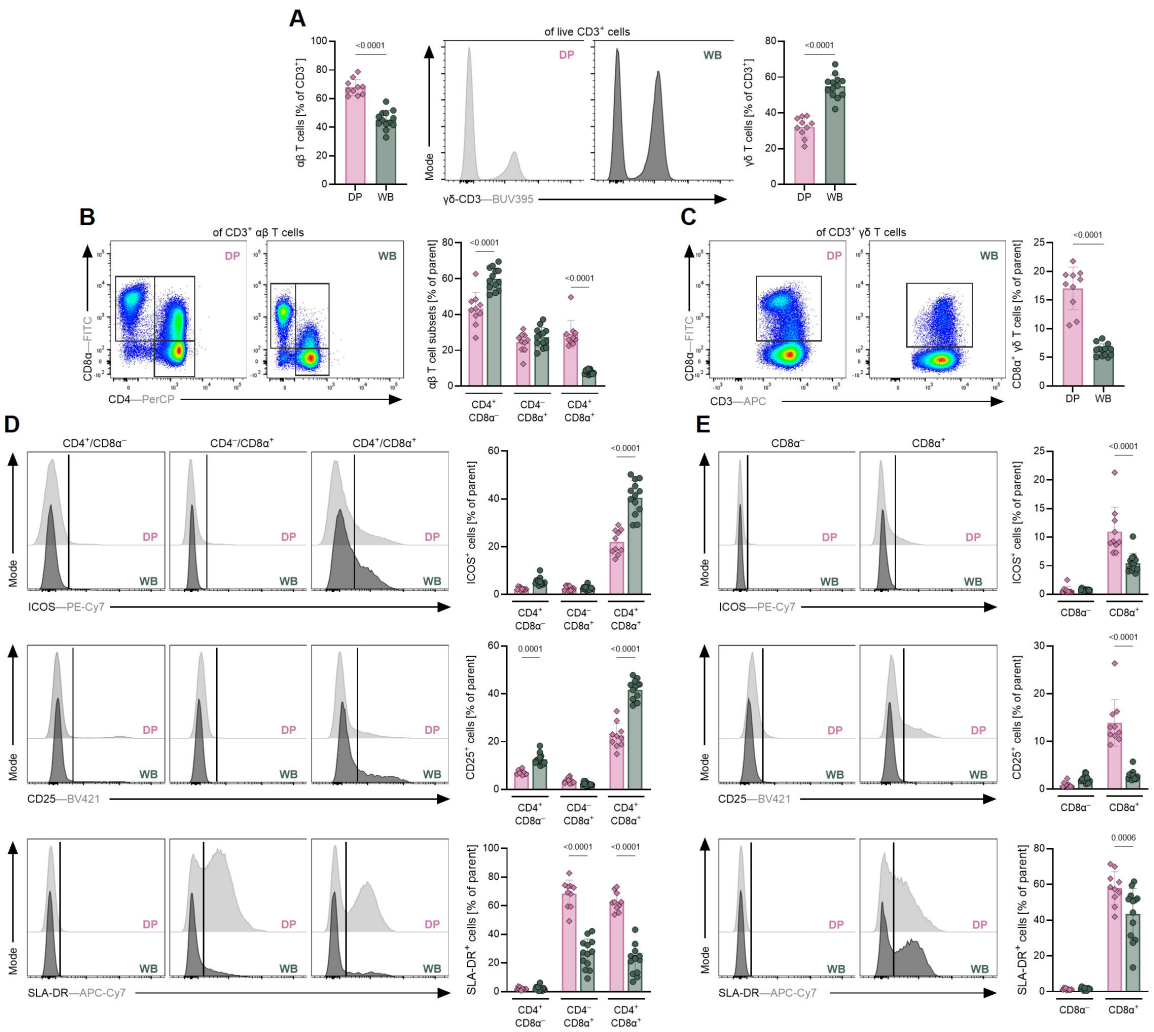
Comparative analysis of T cell populations and their activation status in domestic pig and wild boar. Freshly isolated PBMCs from domestic pigs (DP, *n* = 10, pink squares) and wild boar (WB, *n* = 13, green circles) were investigated by multiparametric flow cytometry. **(A)** Representative histograms show γδCD3 expression of live, single CD3^+^ leukocytes in DP (left, light grey) and WB (right, dark grey). Numbers indicate frequencies in the respective gates. Bar graphs show frequencies of αβ (left) and γδ (right) T cells (DP, pink; WB, green). **(B)** Representative plots show expression of CD4 and CD8α in DP (left) and WB (right). Bar graphs show summarized frequencies of CD4^+^CD8α^−^ (left), CD4^−^CD8α^+^ (middle), and CD4^+^CD8α^+^ (right) cells among αβ T cells. **(C)** Representative plots show expression of CD8α among γδ T cells of DP (left) and WB (right) with bar graphs showing summarized results. **(D)** Representative histograms and bar graphs showing expression of the inducible T-cell co-stimulator (ICOS, top row), CD25 (middle row), and SLA-DR (bottom row) in the indicated αβ T cell populations. **(E)** Representative histograms and bar graphs showing expression of ICOS (top row), CD25 (middle row), and SLA-DR (bottom row) in CD8α^−^ and CD8α^+^ γδ T cell populations. Graphs show mean (SD). Statistical analysis by unpaired t-tests (A, C) or ordinary one-way ANOVA with Holm-Šídák correction (B, D, E). *p* is indicated for all statistically significant differences.

We found a significantly expanded population of γδ T cells in wild boar compared to domestic pigs (**Figure 2A**). Looking at αβ T cell subpopulations, we found markedly fewer differentiated CD4^+^CD8α^+^ and, consequentially, more CD4^+^CD8α^−^ αβ T cells in wild boar, but no differences in CD4^−^CD8α^+^ frequencies. (**Figure 2B**). Among γδ T cells, we found reduced frequencies of CD8α^+^ γδ T cells in wild boar (**Figure 2C**). While still clearly distinct, the expression of CD8α and CD4 was generally lower on lymphocytes from wild boar than domestic pigs (**Figures 2B, C**). Furthermore, we investigated the expression of markers that are generally associated with activation and maturation in porcine lymphocytes: ICOS (inducible T cell co-stimulator), CD25, and SLA-DR (5, 60–62). On αβ T cells, we found higher frequencies of ICOS^+^ and CD25^+^ cells, especially among CD4^+^CD8α^+^ cells in wild boar, while SLA-DR was expressed on more CD4^−^/CD8α^+^ and CD4^+^CD8α^+^ cells in domestic pigs (**Figure 2D**). On γδ T cells, CD8α^+^ cells were the only ones expressing any of the markers, all of which were expressed in higher frequencies in cells from domestic pigs (**Figure 2E**).

### 3.3 Differentiation of porcine memory T cells by multiparametric flow cytometry

**Figure 3:**
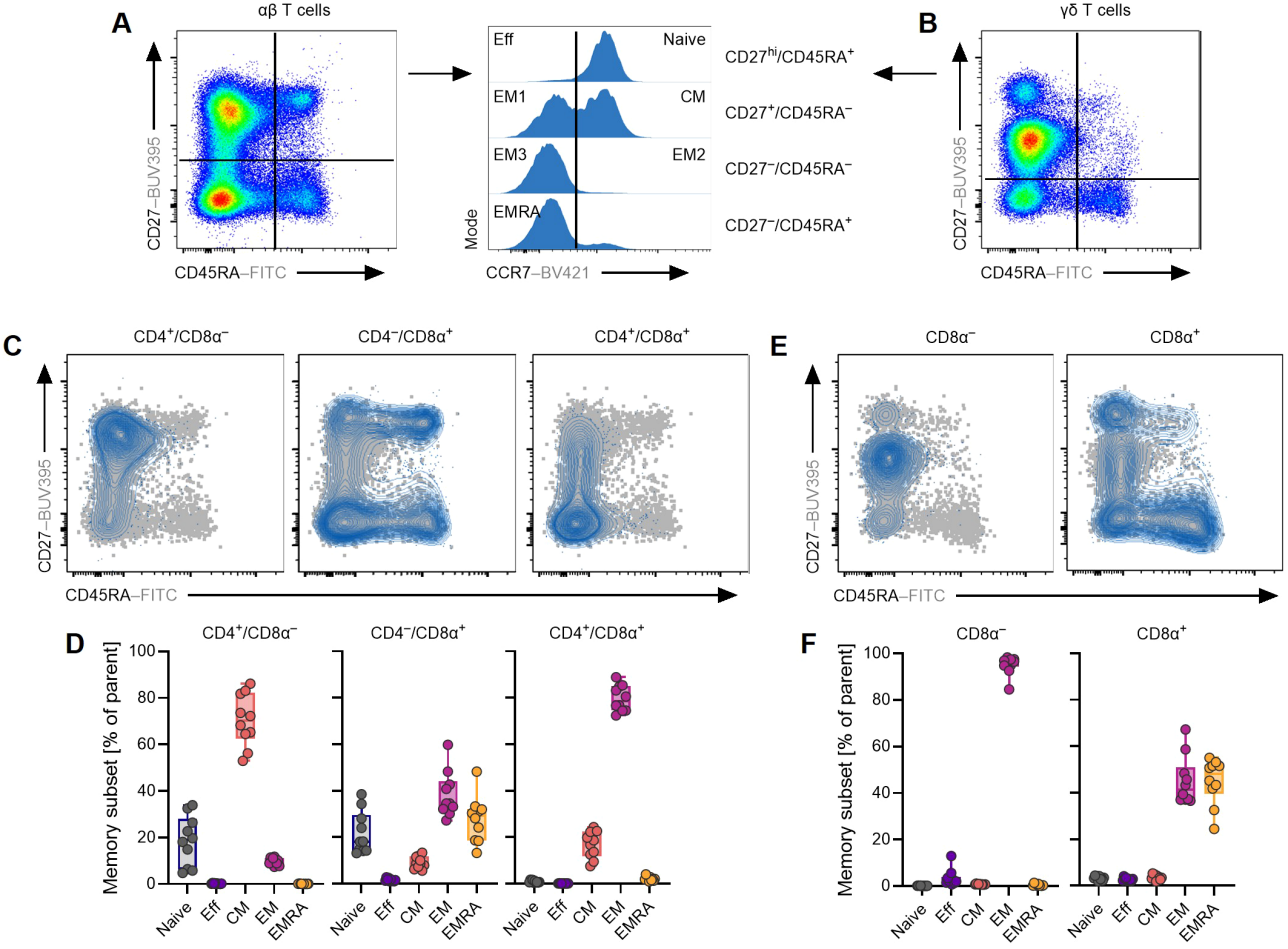
Identification of porcine memory T cells by multiparametric flow cytometry. Freshly isolated PBMCs from domestic pigs (*n* = 10) were investigated by multiparametric flow cytometry. Representative plot shows gating based on CD27 and CD45RA in **(A)** αβ T cells and **(B)** γδ T cells. The histogram shows consecutive gating based on CCR7 expression and identification of the indicated memory T cells populations (see text for detailed information). **(C)** Representative plots show all αβ T cells in grey and subpopulations of CD4^+^CD8α^−^ (left), CD4^−^CD8α^+^ (middle), and CD4^+^CD8α^+^ (right) αβ T cells superimposed in blue. **(D)** Box plots show frequencies of memory T cell populations in CD4^+^CD8α^−^ (left), CD4^−^CD8α^+^ (middle), and CD4^+^CD8α^+^ (right) αβ T cells. **(E)** Representative plots show all γδ T cells in grey and subpopulations of CD8α^−^ (left) CD8α^+^ (right) cells superimposed in blue. **(F)** Box plots show frequencies of memory T cell populations in CD8α^−^ (left) CD8α^+^ (right) γδ T cells. Eff, early effector cells; EM, effector memory cells; CM, central memory cells; EMRA, effector memory cells with re-expression of CD45RA.

To achieve reliable identification of porcine memory T cells by multiparametric flow cytometry, we established an analysis platform by adapting protocols for human T cells. We set up a gating strategy based on expression of CD45RA, CD27, and CCR7 or CD62L (see **Supplementary Figures 1D, E**). The previously mentioned conventional αβ and γδ T cells subpopulations were thus further characterized as belonging to one of four major populations: CD45RA^+^CD27^+^, CD45RA^−^CD27^+^, CD45RA^−^CD27^−^, and CD45RA^+^CD27^−^ in αβ (**Figure 3A**) and γδ T cells (**Figure 3B**). These populations were further investigated for expression of CCR7. Antibodies against CD62L were later added to this panel and could be used interchangeably with CCR7 (**Supplementary Figures 1D, E**). Ultimately, this resulted in five populations phenotypically reflecting human memory T cells, that were termed accordingly: naïve T cells (CD45RA^+^CD27^+^CCR7^+^CD62L^+^), early effector T cells (Eff, CD45RA^+^CD27^+^CCR7^−^CD62L^−^), central memory T cells (CM, CD45RA^−^CD27^+^CCR7^+^CD62L^+^), effector memory T cells (EM, CD45RA^−^CD27^+/−^CCR7^−^CD62L^−^), and terminally differentiated effector memory T cells with re-expression of CD45RA (EMRA, CD45RA^+^CD27^−^CCR7^−^CD62L^−^). The EM populations could be further divided into three subsets, depending on their CD27 and CCR7/CD62L expression (**Figures 3A, B**; **Supplementary Figure 2**). The vast majority of cells belonged to the CD62L^−^CCR7^−^ EM1 or EM3 subsets, while a minor population, EM2, retained CD62L/CCR7 expression. However, EM2 only accounted for small frequencies among all investigated αβ and γδ T cells populations.

To verify the gating and further characterize the memory subpopulations, we first applied this gating strategy to the analysis of peripheral lymphocytes from the cohort of healthy adult domestic pigs (**Figures 3C−F**). Among αβ T cells, the majority of peripheral CD4^+^CD8α^−^ cells belonged to the CM subset, with a considerable naïve population and few further EM. In contrast, CD4^−^CD8α^+^ cells were mostly EM cells with a considerable population of EMRA cells, but also retained a phenotypically naïve population in circulation. In accordance with previous publications, all CD4^+^CD8α^+^ cells displayed an antigen-experienced phenotype, consisting of mostly EM and fewer CM cells. Interestingly and in stark contrast to conventional αβ T cells, the vast majority of γδ T cells exhibited an antigen-experienced phenotype, as we found no phenotypically naïve cells among them (**Figures 3E, F**). Virtually all CD8α^−^ γδ T cells belonged to the EM subset, the more differentiated CD8α^+^ γδ T cells also had a significant population of EMRA cells, corresponding to but exceeding those of their CD8α^+^ αβ TCR counterparts.

### 3.4 Cells with a memory phenotype express high levels of canonical maturation markers

**Figure 4:**
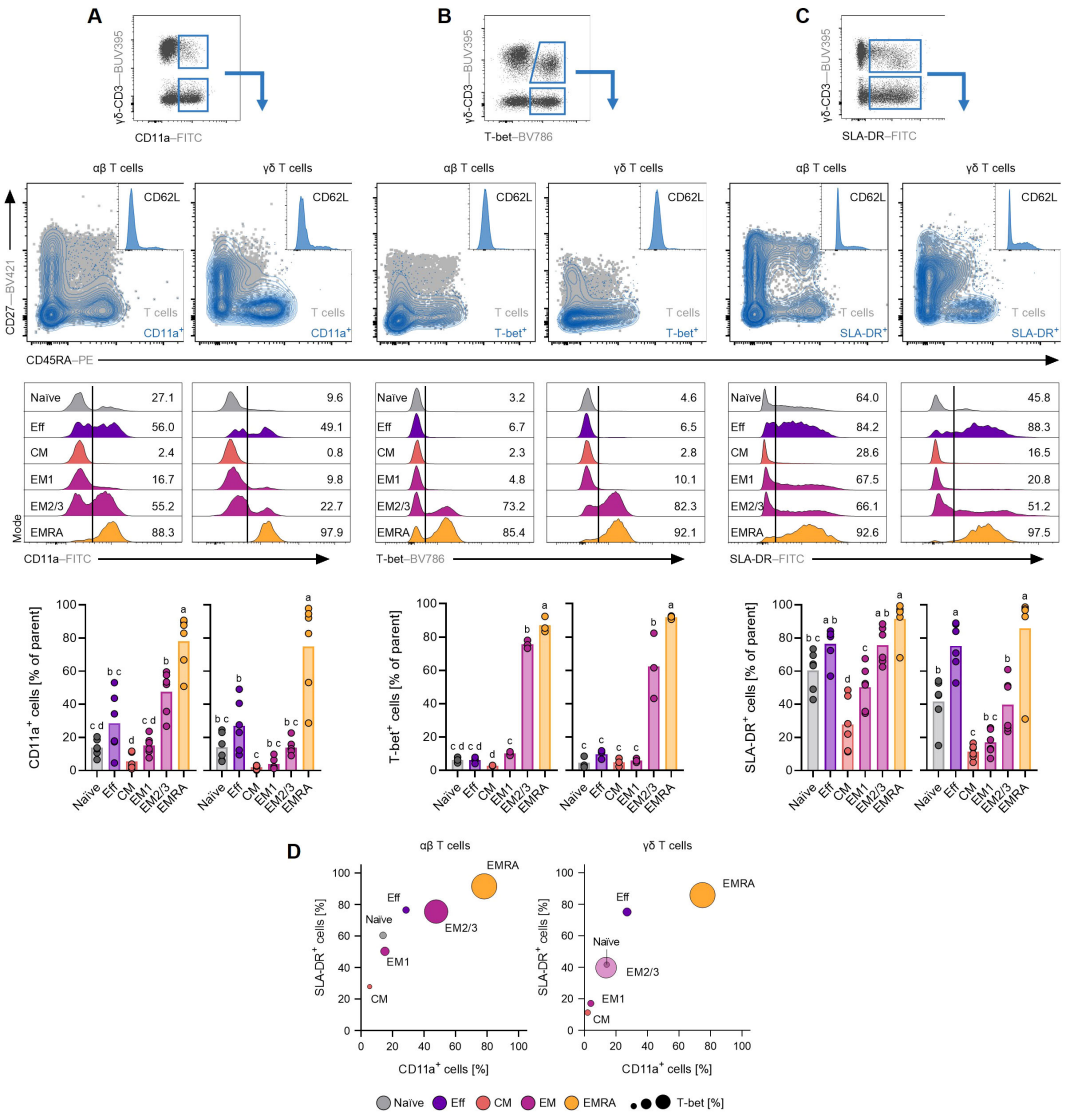
Expression of maturation-associated markers on memory T cells identified by multiparametric flow cytometry. Freshly isolated PBMCs from adult domestic pigs (*n* = 3-6) were investigated for expression of **(A)** CD11a, **(B)** T-bet, and **(C)** SLA-DR on αβ and γδ T cells identified as previously described, without differentiation of CD4^+^ or CD8α^+^ subpopulations. Cells expressing the respective markers were identified as shown in the plots in the top row. Below, marker-positive cells are shown in blue superimposed on all αβ (left) or γδ (right) T cells in grey. Histograms and bar graphs show marker expression and frequencies of expressing cells in memory populations. Numbers in histograms indicate frequencies in the respective positive gates. **(D)** Summarized analysis of marker expression in memory subsets of αβ (left) and γδ (right) T cells. Graphs show mean (SD). Statistical analysis by ordinary one-way ANOVA with Holm-Šídák correction. Compact letter display indicates results of statistical analyses, with different letters indicating statistical significance (*p* < 0.05) between groups. Eff, early effector cells; EM, effector memory cells; CM, central memory cells; EMRA, effector memory cells with re-expression of CD45RA.

Memory T cells differentiate and mature after antigenic activation, which is associated with changes in the expression of various markers. Among those detectable by flow cytometry are integrin CD11a (also known as lymphocyte function-associated antigen 1, LFA-1), T-box transcription factor *TBX21* (T-bet), and SLA-DR (5, 62–65). Expression of these markers was investigated to corroborate our memory gating strategy (dot plots in **Figures 4A–C**, marker-positive cells in blue).

Irrespective of co-receptor expression and TCR chain composition, all investigated activation-associated molecules were expressed at highest levels on EM2/3 and EMRA cells. However, SLA-DR was also expressed on a substantial population of naïve CD45RA^+^CD62L^+^ T cells. We obtained consistent results when T cells were first classified by their memory phenotype and the resulting subpopulations were subsequently analyzed for expression of the respective markers (histograms in **Figures 4A−C**). Generally, a significant expression of all markers was found on late-stage EM and EMRA. In contrast, phenotypically naïve cells did not show substantial expression of the investigated markers, except for SLA-DR. We also found marker-positive cells among presumably early activated Eff cells. Since these cells were relatively scarce, they were not obvious in the dot plots. None of the investigated markers was expressed in substantial levels on CM cells. When the subsets were analyzed based on their respective expression of all markers, naïve and early differentiated subsets clustered predominantly with each other, while late differentiated subsets, i.e., EM2/3 and EMRA cells, were more separated (**Figure 4D**). Overall, separation was more distinct in αβ T cells.

### 3.5 Identified porcine memory T cells express genes associated with their respective differentiation status

**Figure 5:**
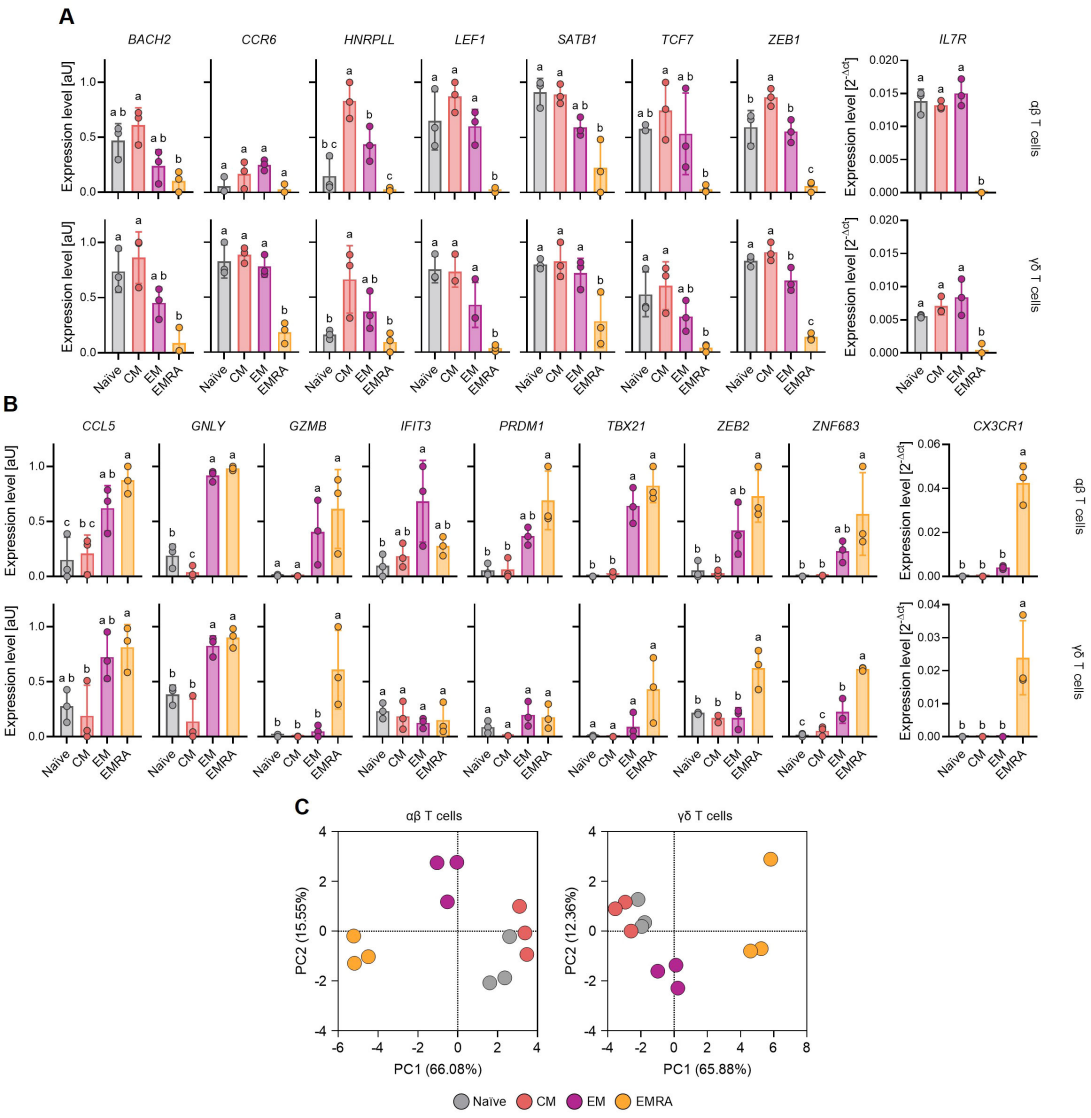
Expression of genes associated with different maturation stages in porcine memory T cell populations. Freshly isolated PBMCs from adult domestic pigs (*n* = 3) were stained according to **Table 1** for cell sorting. Cells were identified as naïve, central memory (CM), effector memory (EM), or terminally differentiated effector memory cells (EMRA) and separated by cell sorting. Sorted cells were lysed and subjected to mRNA extraction and investigation of marker expression by PCR or qPCR. **(A)** Markers associated with naïve status or early maturation or **(B)** antigen experience and late maturation within αβ (top rows) or γδ (lower rows) T cells. Data are normalized for each target. **(C)** Principal component analysis of all markers. Graphs show mean (SD). Statistical analysis by ordinary one-way ANOVA with Holm-Šídák correction. Compact letter display indicates results of statistical analyses, with different letters indicating statistical significance (*p* < 0.05) between groups within each graph.

For more detailed insights on differentiation and maturation, we sorted the four major identified memory populations (naïve, EM, CM, EMRA) of αβ and γδ T cells irrespective of coreceptor expression and investigated the expression levels of various genes that are related to early or late differentiation and maturation of T cells (see **Table 2**) by agarose gel-based PCR and quantitative real-time PCR (qPCR). Markers associated with antigen inexperience or early differentiation were mostly expressed in naïve or CM cells, to a lesser extent in EM, and were not found in EMRA cells (**Figure 5A**). In contrast, markers associated with antigen-experience and later differentiation status were mostly found in EM and EMRA cells, with the highest expression levels in EMRA cells (**Figure 5B**). A principal component analysis based on this gene expression found a clear separation of late differentiated EM and EMRA from each other and both from naïve and early CM subsets (**Figure 5C**).

### 3.6 Cells with a memory phenotype respond faster to *in vitro* activation

**Figure 6:**
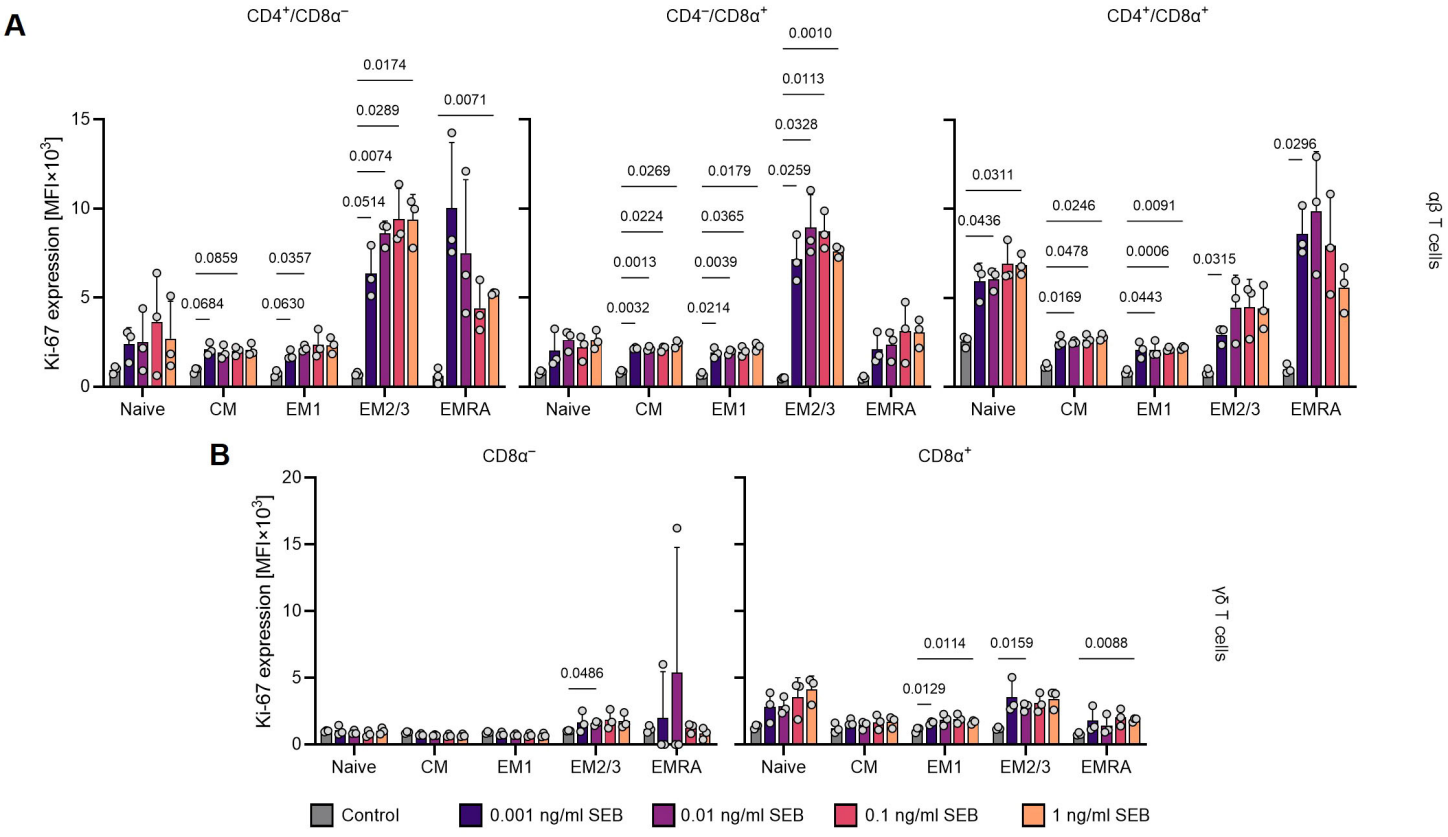
Proliferative responses of porcine memory T cell populations to TCR-dependent stimulation. Freshly isolated PBMCs from adult domestic pigs (*n* = 3) were stimulated with increasing concentrations (0.001−1 ng/ml) of SEB to mimic TCR-dependent activation *in vitro*. After five days of incubation, cells were stained for flow cytometry and proliferation was assessed based on expression of Ki-67. Proliferation measured as mean fluorescence intensity (MFI) of Ki-67 of **(A)** αβ T cell subsets and **(B)** γδ T cell subsets. Bars indicate mean (SD), grey dots show individual data. Statistical analysis by two-way ANOVA with Holm-Šídák correction. *p* is indicated for all statistically significant differences.

A central feature of T cell memory responses is their enhanced responsiveness to activation and TCR stimulation. *In vitro*, mitogens are commonly used to bypass low antigen-specific T cell frequencies and eliminate the need for animal testing. Non-specific mitogens, like PMA (usually in conjunction with ionomycin), activate cells via calcium influx or protein kinase C (66, 67). Concanavalin A (ConA) crosslinks membrane proteins, including the TCR, in *cis* (68), while staphylococcal enterotoxin B (SEB) facilitates binding of MHC and TCR in *trans* (69). Therefore, we used SEB to induce robust and detectable T cell activation while preserving the most natural activation mechanism (**Figure 6**).

**Figure 7:**
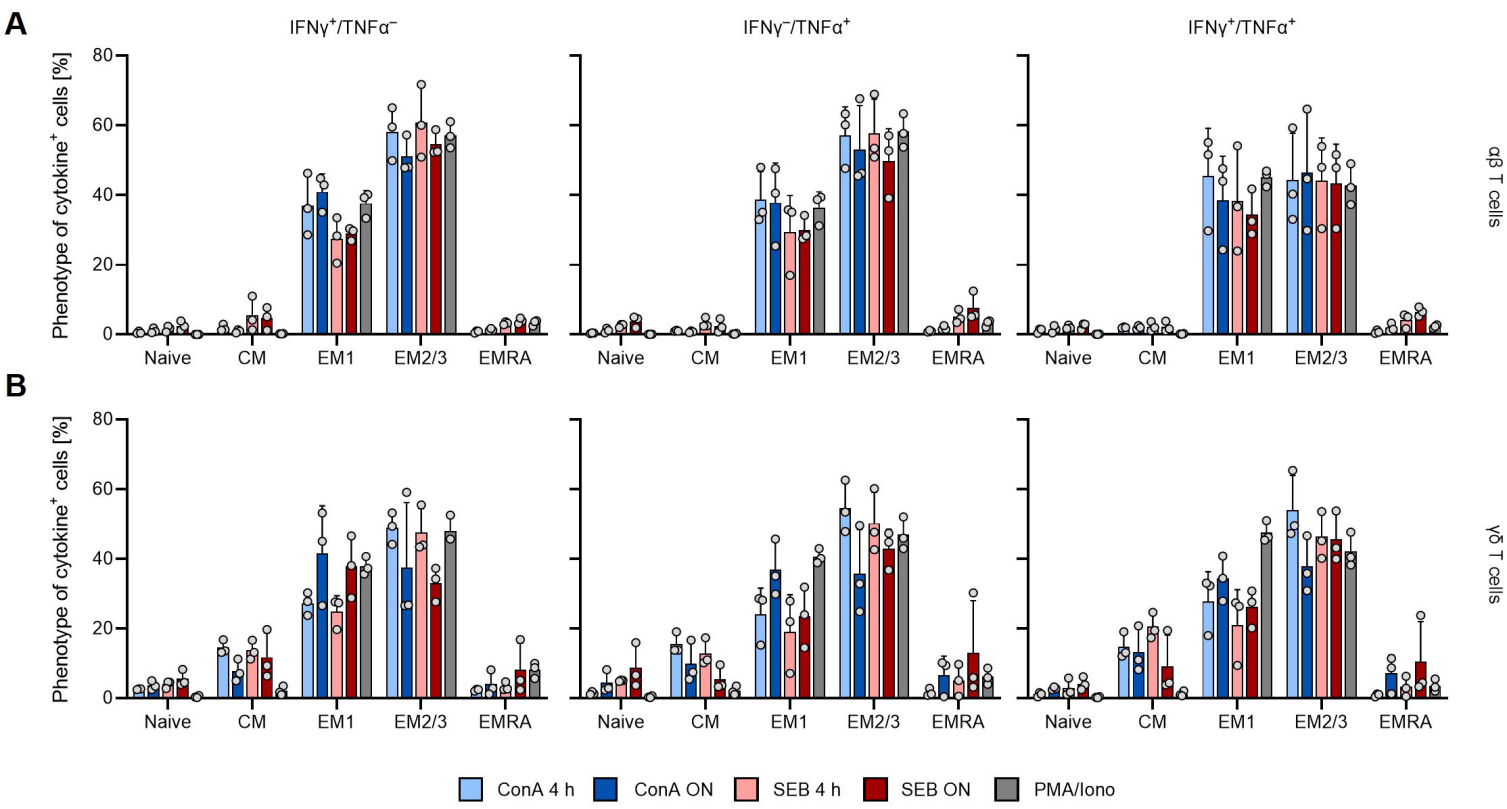
Phenotype of cytokine-secreting T cells after short- or medium-term *in vitro* stimulation. Freshly isolated PBMCs from adult domestic pigs (*n* = 3) were stimulated with ConA (10 µg/ml) or SEB (100 ng/ml) for four hours or stimulated overnight (ON) with restimulation for the last four hours in the morning. PMA/ionomycin (PMA/Iono, 50 ng/ml and 1 µg/ml) was used as a positive control only for the last four hours of incubation. Brefeldin A was added to all conditions for the last four hours of incubation. Cytokine-secreting cells among **(A)** αβ and **(B)** γδ T cell were investigated for their memory phenotype irrespective of CD4 or CD8α expression. Bars indicate mean (SD), grey dots show individual data.

Analogous to proliferative responses, antigen-experienced memory cells show heightened effector functions compared to antigen-inexperienced cells. To investigate cytokine responses in porcine memory T cells, we stimulated PBMCs *in vitro* with ConA and SEB and analyzed the phenotype of cytokine-secreting cells after short-term (4 h) or medium-term (16 h) activation using intracellular cytokine staining (**Figure 7**). Cells that expressed IFNγ and/or TNFα were analyzed for their memory phenotype, regardless of CD4/CD8α co-receptor expression. Similar to the proliferation assay, and irrespective of stimulant and activation period, most cytokine-positive cells phenotypically belonged to the EM subset, both in αβ (**Figure 7A**) as well as γδ (**Figure 7B**) T cells.

### 3.7 Late differentiated memory T cells are less frequent in wild boar than domestic pigs

**Figure 8:**
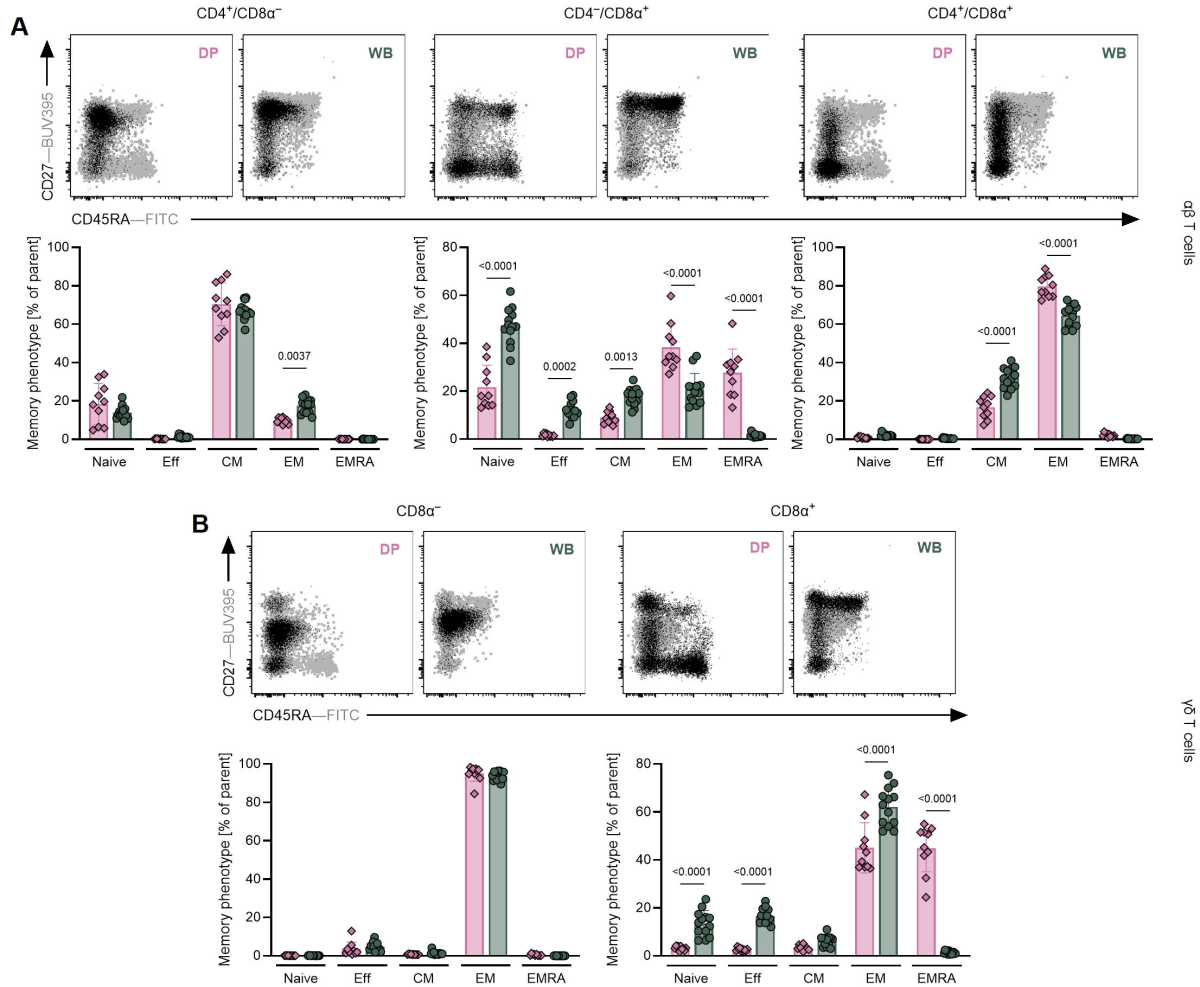
Comparative analysis of memory T cell populations in wild boar and domestic pig. Freshly isolated PBMCs from domestic pigs (DP, *n* = 10, pink squares) and wild boar (WB, *n* = 13, green circles) were investigated for memory phenotype by multiparametric flow cytometry. **(A)** Representative plots show the phenotype of CD4^+^CD8α^−^ (left), CD4^−^CD8α^+^ (middle), and CD4^+^CD8α^+^ (right) αβ T cells in black superimposed on all αβ T cells in grey of DP and WB. Graphs below show frequencies of all identified memory populations in DP (pink) and WB (green). **(B)** Representative plots show the phenotype of CD8α^−^ (left) and CD8α^+^ (right) γδ T cells in black superimposed on all γδ T cells in grey of DP and WB. Graphs below show frequencies of all identified memory populations in DP and WB. Graphs show mean (SD). Statistical analysis by ordinary one-way ANOVA with Holm-Šídák correction. *p* is indicated for all statistically significant differences. Eff, early effector cells; EM, effector memory cells; CM, central memory cells; EMRA, effector memory cells with re-expression of CD45RA.

We employed the established analysis platform and compared lymphocytes from healthy, age-matched wild boar and domestic pigs (**Figure 8**). Lymphocytes from wild boar appeared less differentiated than those in domestic pigs, i.e., there were more naïve and early differentiated cells. This was most obvious among CD4^−^CD8α^+^ cells in wild boar, which had a significantly larger population of naïve cells and fewer EM cells (**Figure 8A**). Among γδ T cells (**Figure 8B**), we found comparable frequencies in CD8α^−^ cells but substantial differences in CD8α^+^ cells. Wild boar had significantly more phenotypically naïve and early differentiated γδ T cells. Most strikingly, we found no late differentiated EMRA cells in wild boar, neither among αβ nor γδ T cells.

Overall, we found substantial differences in the leukocyte and T cell landscape of wild boar and domestic pigs. When applying our newly established T cell phenotyping strategy, we found significantly fewer differentiated cells in wild boar than in domestic pigs, which was most prominent in the complete lack of late differentiated EMRA cells in wild boar. However, while less differentiated, the remaining cells expressed activation markers in comparable or even higher levels than their counterparts in domestic pigs.

## 4 Discussion

The continuing spread of high-impact pathogens like African swine fever virus (ASFV) poses a global threat for both animal conservation and economic interests alike. Vaccination would aid disease management but its success depends on a thorough understanding of the porcine immune system, especially of wild boar. Analyzing the memory immune response is especially important for uncovering potential species- and breed-specific differences in antiviral protection and duration of immunity following vaccination or infection. Using a comparative approach, we found significant variations in leukocyte populations of domestic pigs and age-matched wild boar. Using markers commonly used in human studies, we characterized memory T cell subsets in pigs analogous to those in humans and found that wild boar exhibited fewer differentiated cells, which nevertheless remain highly activated.

Despite similar genetic backgrounds of domestic pigs and Eurasian wild boar, significant phenotypic differences emerge due to the influence of nutritional and environmental factors impacting epigenetic modulations (72). In wild boar, we found fewer antigen-presenting cells expressing SLA-DR, also at lower levels (measured by MFI). Interestingly, cells of the innate immune system, i.e., PMN and NK cells, were also reduced in frequency in wild boar. This was usually not found in previous comparative studies of wild and laboratory rodents (11, 15), which might indicate effects specific for pigs. These cells might also be significantly influenced by seasonal changes, which have been shown to influence immune cell frequencies and effector functions as wells as other physiological parameters in wild rodents (73–75). Similar responses to seasonal changes have also been shown for vertebrates (76). Whether the differences described in this study can be attributed to comparable effects remains unknown and warrants further investigations, e.g., during other seasons.

The T cell pool of wild boar in our study was characterized by reduced frequencies of differentiated cells. Though less abundant, these cells appeared highly activated, suggesting a trade-off in immune activation of wild animals. A higher expression of activation markers on lymphocytes of wild animals, often concurring with reduced cellular responses at least upon *in vitro* stimulation, has been observed in wild rodents before (11, 14, 15), but was also shown in a single-cell analysis of jejunal lymphocytes for Asian wild boar (77). It was hypothesized that this apparent discrepancy is the result of an increased microbial burden in wild animals compared to their domesticated counterparts (11). Wild boar often harbor parasites that, while not life-threatening by themselves, are energetically costly (78). Interestingly, the daily energy expenditure does not increase upon parasite infection, indicating compensation by reduction of host processes, including immunity (79), which might also affect the wild boar in this study. In addition (and unlike domestic pigs), wild boar constantly forage for food and face seasonal shortages, e.g., during winter and early spring (80, 81). This further constraint demands conservation of energy, possibly raising the threshold for immune activation. This leads to narrowed responses and, ultimately, fewer differentiated cells, as indicated by previous studies, mainly in mice (82, 83). While the animals in this study did not face shortages, these constraints might be genetically imprinted from their parents, as has been shown previously in humans (84). However, a larger naïve T cell population is also indicative of an increased ability to mount responses against new antigens (85, 86). The findings of this study might contribute to explaining previously observed differences between domestic pigs and wild boar in experimental infection studies with ASFV. While both conspecifics are susceptible to infection with highly virulent ASFV (19), wild boar tend to succumb earlier after infection and have higher fatality rates even when infected with moderately virulent ASFV compared to domestic pigs that typically survive infection with these strains (16–18, 87). Based on our hypothesis of a higher immune activation threshold, the increased susceptibility might be explained by responses that are initiated too late, ultimately being overwhelmed by the systemic viral infection (as indicated by significantly higher viral loads in ASFV-infected wild boar (20)). These delayed (and consequently impaired) responses might cause immunopathology, a phenomenon increasingly recognized to result not only from excessive immune responses but also from impaired or dysregulated immunity. For example, reduced viral clearance prolongs and enhances T cell stimulation by antigen-presenting cells, which in turn leads to uncontrolled T cell activation, heightened secretion of pro-inflammatory cytokines, and impaired regulatory responses (88). However, while comparative pathological studies confirmed higher susceptibility and mortality of ASFV-infected wild boar, a distinct pathological explanation for these differences has not yet been found (20, 87).

Using the antibody panel and gating strategy described in this study, we were able to phenotypically identify memory T cell populations that were not described previously or only to a limited extent. The employed strategy is based on previous publications in humans (26, 27) but also studies of memory responses of porcine T cells that used at least some of the markers (2, 31–39). Thus, our staining protocol is consistent with but expands previous efforts. Of note, our data challenge the prevailing notion that memory formation of porcine T helper cells requires co-expression of CD8α, which has hitherto been considered the main indicator of a memory phenotype. In contrast, our data indicate that while indeed all CD4^+^CD8α^+^ T cells belong to memory subsets, a substantial population among CD4^+^CD8α^−^ T cells is also antigen-experienced, at least phenotypically. This observation also highlights the necessity of more extensive marker panels to accurately investigate porcine T cell memory.

Memory responses in γδ T cells are less well characterized than in conventional αβ T cells, especially in pigs. While human γδ T cells mount memory responses and follow differentiation patterns involving CD45RA and CD27 (89–91), responses of porcine γδ T cells remain largely unexplored. However, some studies obtained evidence of memory formation in porcine γδ T cells: Porcine reproductive and respiratory syndrome virus infection induced antigen-specific cytokine expression and proliferation in γδ T cells, which also showed recall responses after re-exposure, indicating memory formation (92, 93). Vaccination with an H3N2 swine influenza virus induced similar responses after immunization and challenge (94). However, neither of these studies investigated expression of memory markers on those γδ T cells. Still, given the high degree of resemblance of immune cell populations and mechanisms between humans and pigs (1–6), it seems reasonable to assume that porcine γδ T cells follow similar differentiation patterns and express similar molecules as their human counterparts, thus overall supporting the gating strategy presented in this study. Contrasting the assumption that CD8α expression on γδ T cells is associated with antigen experience, effector function, and differentiation (95–98), we also found some phenotypically naïve cells among CD8α^+^ γδ T cells in wild boar. While the true nature of these cells remains to be elucidated, a previous study in humans has found memory T cells with a naïve phenotype (99).

Our data also call for cautious analysis of memory responses in the context of vaccination or infection, e.g., for the identification of correlates of protection. Focus on major subsets, i.e., CD4^+^CD8α^−^, CD4^−^CD8α^+^, and CD4^+^CD8α^+^ T cells, in restimulation assays without comprehensive identification of the responding memory T cell subset might hinder detection of relevant activation patterns. Instead, the maturation stage of the cells expressing the cytokines might be pivotal. If an infection or immunization induces responses of naïve or early differentiated T cells but fails to induce differentiation into late-stage memory cells, the protective benefit might be limited. In contrast, detection of responses in differentiated subsets (like late-stage EMs or EMRAs) could indicate more robust protection, especially against pathogens with fast reproduction, like viruses (100). Slower pathogens, though, might be better fought off by other memory subsets, like CMs (100). Long-lasting, protective responses correlating with induction of late-stage differentiated EMRA cells have also been shown in veterinary species, for example in dogs after vaccination against rabies virus (101). This is particularly important considering diseases where protection is heavily dependent on T cell responses, i.e., ASFV (21), which in addition is difficult to use in oral vaccination efforts needed for wild boar (102). However, these analyses necessitate more advanced technical equipment, i.e., flow cytometers or spectral analyzers with a high number of distinguishable fluorochromes, which might not be readily available at every institution.

Identification of memory subsets solely based on phenotyping has its limitations, as the strict cutoffs used in methods like flow cytometry are unable to accurately reflect the exact cell identity. This is particularly important for markers with a gradient expression, like CCR7, CD26L, and CD27 (103). Accordingly, it remains unknown whether the three EM subsets we have described in this study are an accurate account of their memory state. However, cells with the same phenotype have also been reported in humans (26). Moreover, EM cells with residual expression of CD62L or CCR7 and, thus, a phenotypical overlap between EM and CM, have been reported and proposed as “peripheral memory” cells that are able to circulate between lymphoid and non-lymphoid tissues while maintaining effector memory functions (104). We observed reduced cytokine responses in EMRA cells, which might be surprising, given their presumed high effector functions. However, this can be explained by two factors: First, EMRA are one of the smallest subsets and even high responses would be diminished by other, larger subsets. Second, cytokine responses often peak in EM cells rather than EMRAs (105). Nevertheless, this could also be an indication of immunosenescence, that has been associated with an EMRA phenotype in some studies (106).

It should be noted that husbandry conditions in this study, including outdoor access in wild boars and routine vaccination in domestic pigs, while reflective of regular living conditions for these animals, may have contributed to some of the observed differences. These factors are likely to have a more significant impact than the animals’ genomes, as comparative genomic studies found minor differences between wild boar and domestic pigs within the same geographical context, but considerable divergence between European and Asian pigs, likely as a result of independent domestication (70, 71). While investigation of other confounders, like environmental pollutants or stress especially in wild boar, require studies with larger and more diverse groups, our results pave the way for these studies. In addition, the data obtained from domestic pigs refer specifically to the German Landrace breed. Future studies will have to assess how much variation exists among different pig breeds with respect to the lymphocyte populations characterized in this study. Previous studies across breeds of various animals including chicken, sheep, cows, and pigs, have found considerable breed-specific differences in composition and responses of their respective immune systems (107–111). Various studies investigated antiviral immune responses in different pig breeds, mainly against porcine reproductive and respiratory syndrome virus (PRRSV). Highly domesticated breeds like Large White, Landrace, or Duroc were usually more susceptible to PRRSV infection and developed more pronounced clinical disease than older breeds, which was associated with distinct differences in the immune response patterns (112–114). However, similar to our results, a study in Asian wild boar found a higher expression of activation markers in wild boar lymphocytes compared to cells from pig breeds with a higher degree of domestication (77), indicating conserved mechanisms in wild pigs even across geographical regions. Still, further research is needed to elucidate the differences between the immune systems of domestic pigs, including other breeds, and wild boar.

Our study provides novel insights into the immune cell landscape in wild and domestic pigs, providing a foundation for more detailed studies of porcine memory T cell differentiation following vaccination and infection. Notably, the reduced T cell differentiation observed in wild boar demonstrates the influences of species-specific immune traits on immune responses, which has pivotal implications for vaccination strategies. Furthermore, our staining protocols allow for a direct comparison between human and porcine memory T cells alongside deep analysis of swine memory T cells, further supporting pigs as biomedical models and enabling translational insights that can inform both veterinary and human immunology.

## Data Availability

The original contributions presented in the study are included in the article/**Supplementary Material**. Further inquiries can be directed to the corresponding author.
