# Supplementary material for: How the wild things are: comparative T cell phenotyping and memory T cell identification in wild boar and domestic pigs
Source: Front Immunol. 2026 Jun 18;17:1844742. doi: 10.3389/fimmu.2026.1844742 (PMC13322931; doi:10.3389/fimmu.2026.1844742)
Supplement: Supplementary file 1 [file Table1.docx]

# Statistical analyses

**Supplementary Table 1.** Statistical effect sizes for data in Figure 1.

| Comparison | p-value | Cohen’s d | Effect size r |
| --- | --- | --- | --- |
| PMN | <0.0001 | 2.83 | 0.817 |
| Monocytes | 0.2669 | 0.480 | 0.233 |
| Dendritic cells | 0.0002 | 1.917 | 0.692 |
| B cells | 0.0585 | 0.841 | 0.387 |
| T cells | <0.0001 | 3.649 | 0.884 |
| NK cells | 0.0001 | 2.008 | 0.708 |
| SLA-DR^+^ B cells | 0.0430 | 0.910 | 0.414 |
| SLA-DR^+^ monocytes | 0.0065 | 1.270 | 0.536 |
| SLA-DR^+^ DC | 0.0001 | 1.961 | 0.700 |
| SLA-DR expression on B cells | <0.0001 | 2.690 | 0.803 |
| SLA-DR expression on monocytes | <0.0001 | 4.022 | 0.895 |
| SLA-DR expression on DC | <0.0001 | 2.588 | 0.791 |

**Supplementary Table 2.** Statistical effect sizes for data in Figure 2A and 2C.

| Comparison | p-value | Cohen’s d | Effect size r |
| --- | --- | --- | --- |
| αβ T cells | <0.0001 | 3.719 | 0.881 |
| γδ T cells | <0.0001 | 3.730 | 0.881 |
| CD8α^+^ γδ T cells | <0.0001 | 4.194 | 0.903 |

**Supplementary Table 3.** Statistical effect sizes for data in Figure 2B, D, E.

| Comparison | p-value | R^2^ (≙ η^2^) |
| --- | --- | --- |
| αβ T cell subsets | <0.0001 | 0.8901 |
| ICOS^+^ cells (αβ T cells) | <0.0001 | 0.9444 |
| CD25^+^ cells (αβ T cells) | <0.0001 | 0.9619 |
| SLA-DR^+^ cells (αβ T cells) | <0.0001 | 0.9229 |
| ICOS^+^ cells (γδ T cells) | <0.0001 | 0.7891 |
| CD25^+^ cells (γδ T cells) | <0.0001 | 0.8251 |
| SLA-DR^+^ cells (γδ T cells) | <0.0001 | 0.8958 |

**Supplementary Table 4.** Statistical effect sizes for data in Figure 4A-C.

| Comparison | p-value | R^2^ (≙ η^2^) |
| --- | --- | --- |
| CD11a^+^ cells (αβ T cells) | <0.0001 | 0.8399 |
| CD11a^+^ cells (γδ T cells) | <0.0001 | 0.7963 |
| T-bet^+^ cells (αβ T cells) | <0.0001 | 0.9968 |
| T-bet^+^ cells (γδ T cells) | <0.0001 | 0.9630 |
| SLA-DR^+^ cells (αβ T cells) | <0.0001 | 0.7652 |
| SLA-DR^+^ cells (γδ T cells) | <0.0001 | 0.7865 |

**Supplementary Table 5.** Statistical effect sizes for data in Figure 5A.

| Comparison | p-value | R^2^ (≙ η^2^) |
| --- | --- | --- |
| *BACH2* (αβ T cells) | 0.0089 | 0.7474 |
| *BACH2* (γδ T cells) | 0.0030 | 0.8093 |
| *CCR6* (αβ T cells) | 0.0294 | 0.6557 |
| *CCR6* (γδ T cells) | 0.0001 | 0.9130 |
| *HNRPLL* (αβ T cells) | 0.0005 | 0.8805 |
| *HNRPLL* (γδ T cells) | 0.0199 | 0.6890 |
| *LEF1* (αβ T cells) | 0.0014 | 0.8416 |
| *LEF1* (γδ T cells) | 0.0007 | 0.8672 |
| *SATB1* (αβ T cells) | 0.0016 | 0.8383 |
| *SATB1* (γδ T cells) | 0.0105 | 0.7370 |
| *TCF7* (αβ T cells) | 0.0243 | 0.6724 |
| *TCF7* (γδ T cells) | 0.0133 | 0.7203 |
| *ZEB1* (αβ T cells) | <0.0001 | 0.9239 |
| *ZEB1* (γδ T cells) | <0.0001 | 0.9612 |
| *IL7R* (αβ T cells) | <0.0001 | 0.9662 |
| *IL7R* (γδ T cells) | 0.0013 | 0.8452 |

**Supplementary Table 6.** Statistical effect sizes for data in Figure 5B.

| Comparison | p-value | R^2^ (≙ η^2^) |
| --- | --- | --- |
| *CCL5* (αβ T cells) | 0.0032 | 0.8058 |
| *CCL5* (γδ T cells) | 0.0173 | 0.7004 |
| *GNLY* (αβ T cells) | <0.0001 | 0.9851 |
| *GNLY* (γδ T cells) | 0.0002 | 0.9018 |
| *GZMB* (αβ T cells) | 0.0287 | 0.6578 |
| *GZMB* (γδ T cells) | 0.0087 | 0.7492 |
| *IFIT3* (αβ T cells) | 0.0333 | 0.6443 |
| *IFIT3* (γδ T cells) | 0.6382 | 0.1814 |
| *PRDM1* (αβ T cells) | 0.0024 | 0.8204 |
| *PRDM1* (γδ T cells) | 0.0807 | 0.5498 |
| *TBX21* (αβ T cells) | <0.0001 | 0.9419 |
| *TBX21* (γδ T cells) | 0.0323 | 0.6471 |
| *ZEB2* (αβ T cells) | 0.0032 | 0.8058 |
| *ZEB2* (γδ T cells) | 0.0017 | 0.8361 |
| *ZNF683* (αβ T cells) | 0.0220 | 0.6811 |
| *ZNF683* (γδ T cells) | <0.0001 | 0.9622 |
| *CX3CR1* (αβ T cells) | <0.0001 | 0.9588 |
| *CX3CR1* (γδ T cells) | 0.0016 | 0.8373 |

**Supplementary Table 7.** Statistical effect sizes for data in Figure 6.

| Comparison | p-value | Partial η^2^ |
| --- | --- | --- |
| CD4^+^/CD8α^−^ αβ T cells | <0.0001 | 0.6597 |
| CD4^−^/CD8α^+^ αβ T cells | <0.0001 | 0.8231 |
| CD4^+^/CD8α^+^ αβ T cells | <0.0001 | 0.7218 |
| CD8α^−^ γδ T cells | 0.9240 | 0.1706 |
| CD8α^+^ γδ T cells | 0.1053 | 0.3946 |

**Supplementary Table 8.** Statistical effect sizes for data in Figure 8.

| Comparison | p-value | R^2^ (≙ η^2^) |
| --- | --- | --- |
| CD4^+^/CD8α^−^ αβ T cells | <0.0001 | 0.9640 |
| CD4^−^/CD8α^+^ αβ T cells | <0.0001 | 0.8525 |
| CD4^+^/CD8α^+^ αβ T cells | <0.0001 | 0.9854 |
| CD8α^−^ γδ T cells | <0.0001 | 0.9976 |
| CD8α^+^ γδ T cells | <0.0001 | 0.9425 |
