# Supplementary figures and images for: How the wild things are: comparative T cell phenotyping and memory T cell identification in wild boar and domestic pigs
Source: Front Immunol. 2026 Jun 18;17:1844742. doi: 10.3389/fimmu.2026.1844742 (PMC13322931; doi:10.3389/fimmu.2026.1844742)

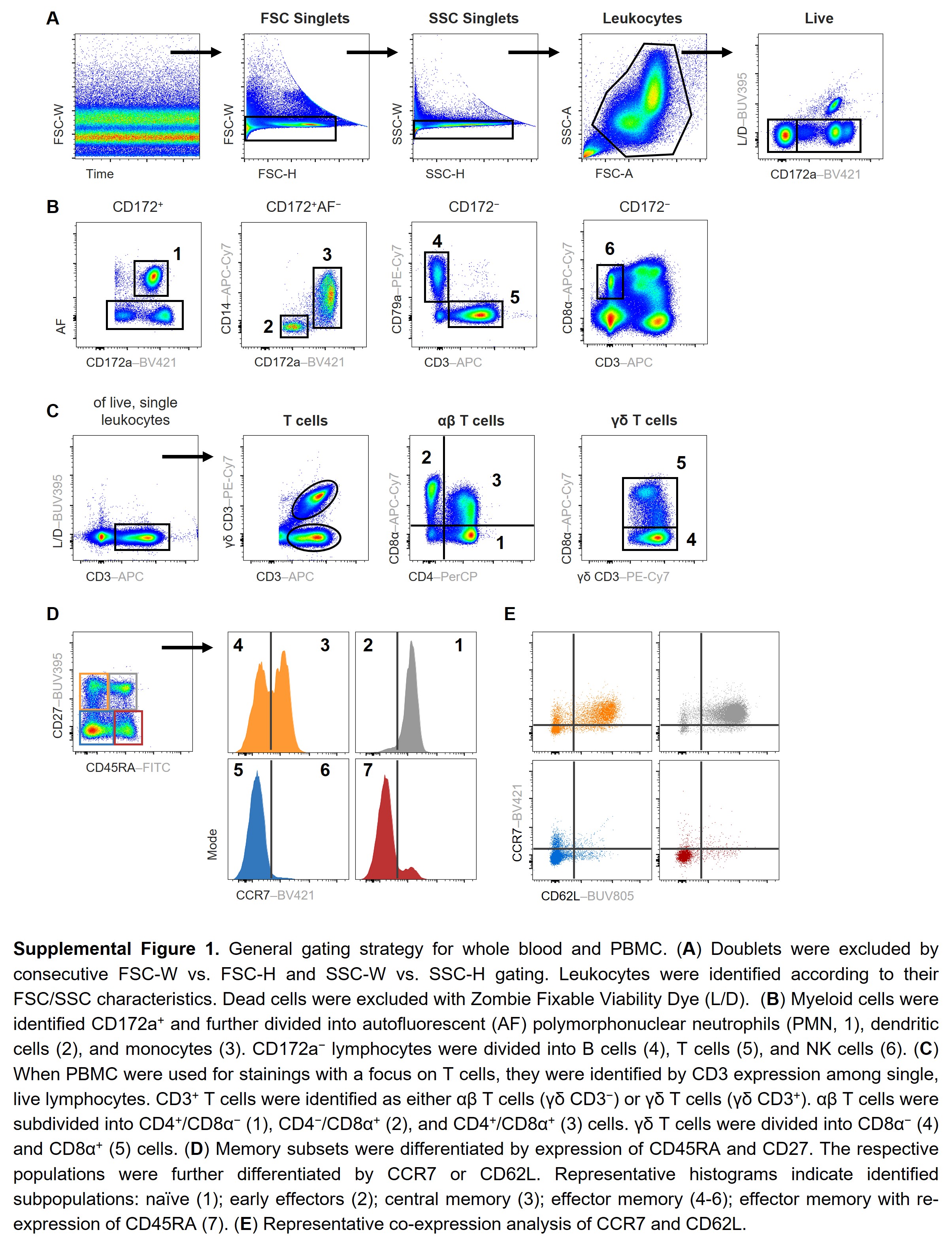

Supplement: Supplementary file 2 [file Image1.jpeg]

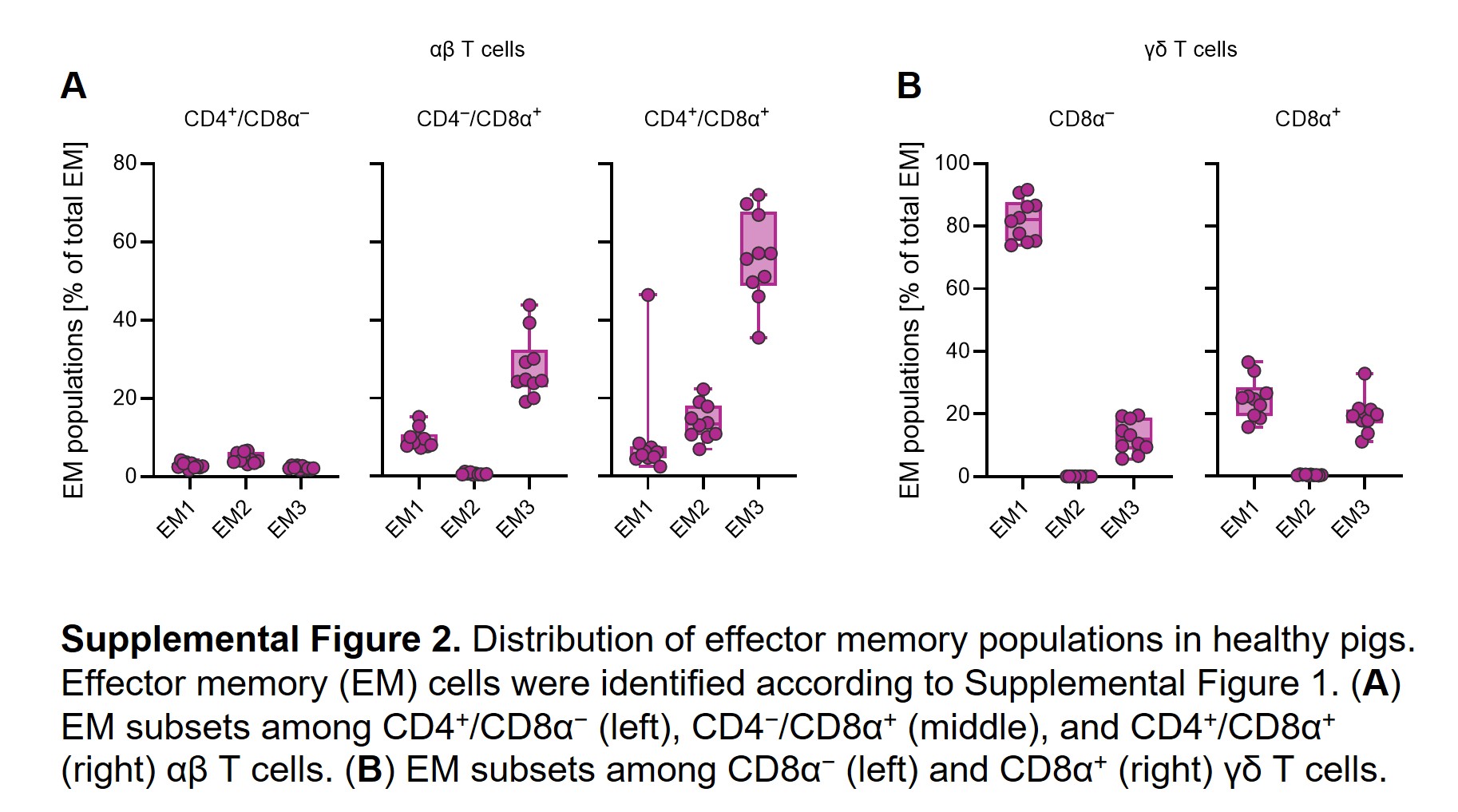

Supplement: Supplementary file 3 [file Image2.jpeg]

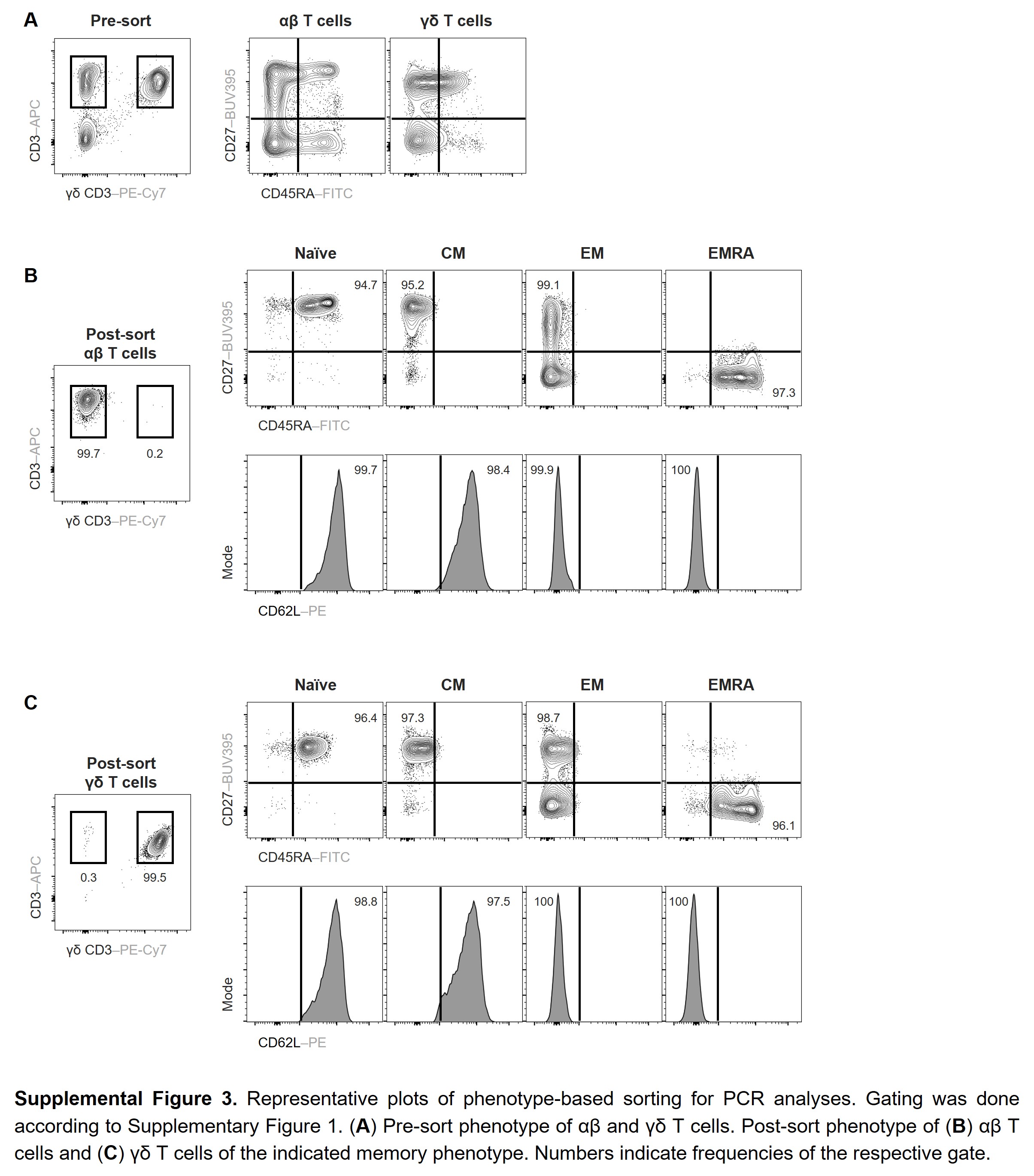

Supplement: Supplementary file 4 [file Image3.jpeg]
